# JNK, p38, ERK, and SGK1 Inhibitors in Cancer

**DOI:** 10.3390/cancers10010001

**Published:** 2017-12-21

**Authors:** Jonas Cicenas, Egle Zalyte, Arnas Rimkus, Dalius Dapkus, Remigijus Noreika, Sigitas Urbonavicius

**Affiliations:** 1 Department for Microbiology, Immunbiology und Genetics, Max F. Perutz Laboratories, University of Vienna, Vienna AT-1030, Austria; 2 Proteomics Centre, Institute of Biochemistry, Vilnius University, 01513 Vilnius, Lithuania; egle.zalyte@gmail.com; 3 MAP Kinase Resource, Bioinformatics, Melchiorstrasse 9, CH-3027 Bern, Switzerland; 4 Faculty of Medicine, Vilnius University, 01513 Vilnius, Lithuania; rimkus.arnas@gmail.com; 5 Department of Biology and Chemistry, Lithuanian University of Educational Sciences, 08106 Vilnius, Lithuania; dalius.dapkus@leu.lt (D.D.); remigijus.noreika@leu.lt (R.N.); 6 Cardiovascular Research Centre, Viborg Hospital, Heibergs Alle 4, 8800 Viborg, Denmark; su@clin.au.dk

**Keywords:** JNK, p38, ERK, SGK1, kinase inhibitors, cancer, MAP kinases

## Abstract

Mitogen-activated protein kinases (MAP kinases) are a family of kinases that regulates a range of biological processes implicated in the response to growth factors like latelet-derived growth factor (PDGF), epidermal growth factor (EGF), vascular endothelial growth factor (VEGF), and stress, such as ultraviolet irradiation, heat shock, and osmotic shock. The MAP kinase family consists of four major subfamilies of related proteins (extracellular regulated kinases 1/2 (ERK1/2), c-Jun N-terminal kinase (JNK), p38, and extracellular regulated kinase 5 (ERK5)) and regulates numerous cellular activities, such as apoptosis, gene expression, mitosis, differentiation, and immune responses. The deregulation of these kinases is shown to be involved in human diseases, such as cancer, immune diseases, inflammation, and neurodegenerative disorders. The awareness of the therapeutic potential of the inhibition of MAP kinases led to a thorough search for small-molecule inhibitors. Here, we discuss some of the most well-known MAP kinase inhibitors and their use in cancer research.

## 1. Introduction

**Figure 1:**
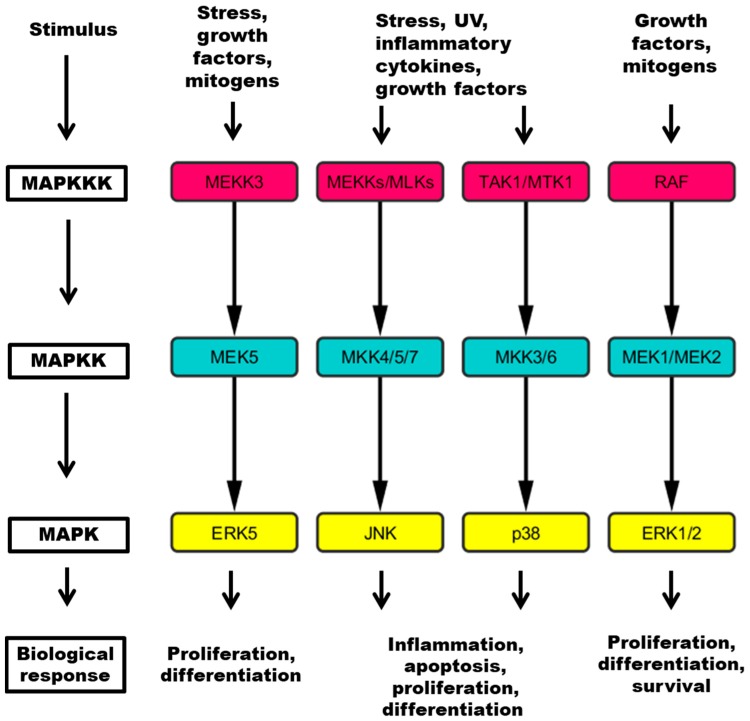
Four major pathways contain a three tiered kinase cascade comprising a MAP kinase kinase kinase (MAPKKK), a MAP kinase kinase (MAPKK) and the MAPK, which mediates responses to specific stimuli. MEKK: mitogen-activated kinase kinase kinase; MLK: mixed lineage kinase; TAK: Tat-associated kinase; MTK: mitogen-activated protein kinase kinase kinase 4; RAF: RAF proto-oncogene serine/threonine kinase; MEK: mitogen-activated protein kinase kinase; MKK: mitogen-activated protein kinase kinase; ERK: extracellular regulated kinase; JNK: c-Jun N-terminal kinase; UV: ultraviolet light.

Protein kinases are a family of enzymes that phosphorylate proteins on serine, threonine, or tyrosine. Protein phosphorylation brings about changes of their functions, such as their interaction with other proteins, localization, or enzymatic activity. Our genome contains more than 500 protein kinase genes as well as some pseudogenes. Protein phosphorylation plays a critical role in the regulation of numerous cellular properties such as proliferation, differentiation, apoptosis, migration, and adhesion. Therefore, the wrong kinase activity can result in exceptional alterations of these processes. In fact, defective kinases are often found to be oncogenic and can be important for the existence of cancer cells. In addition, the phosphorylation of some proteins, such as protein kinase B, also known as Akt (Akt) [1,2], epidermal growth factor (EGF), [3], ErbB receptor family member 2 (ERBB2) [4,5], extracellular regulated kinase (ERK) [6,7], p38 [8], and Src homology 2 domain-containing-transforming protein C-A (SchA) [9], is associated with prognosis in several human cancers. The first MAP kinase network to be discovered was the GTPase Ras-RAF proto-oncogene serine/threonine-protein kinase-extracellular regulated kinase (RAS-RAF-ERK) signal transduction cascade (Figure 1), defined by ERK1 and ERK2 [10,11]. The ERK cascade functions in cellular proliferation, differentiation, and survival and deregulation of it is common in cancer. In many cases, the activity of ERKs in cancer depends on mutations in RAS and RAF kinases [12,13]. c-Jun N-terminal kinase (JNK) is a subfamily of mitogen-activated protein (MAP) (Figure 1) kinases originally identified as kinases that bind and phosphorylate transcription factor AP-1 (JUN) on S63 and S73 within its transcriptional activation domain [14]. There are three differently spliced genes in the subfamily, namely, JNK1, JNK2, and JNK3. Aberrant activation of JNKs is found in many cancers, as well as inflammatory and neurodegenerative disorders. p38 is yet another subfamily (Figure 1), consisting of four isoforms: α, β, γ, and δ [15]. Pathogens or inflammatory stimuli initiate a cascade mediated by p38 kinases and abnormal activity of these kinases is observed in inflammatory diseases and cancers. One of the major substrates and further signal transducers is serum/glucocorticoid-regulated kinase 1 (SGK1) kinase, which is also important in cancer development. The last of four major MAP kinase pathways is the mitogen-activated protein kinase kinase 5- extracellular regulated kinase 5 (MEK5-ERK5) cascade (Figure 1). Activation of this pathway is a common event in tumor development and it is involved in anti-apoptotic signaling and chemoresistance [16]. There are many inhibitors, which are quite specific and quite a few of them are already approved for cancer therapy or at least in clinical studies of different phases. Thus far, several kinases are quite popular targets of inhibition in cancers, such as tyrosine kinases [17], cyclin-dependent kinases (CDKs) [18,19,20] and aurora kinases [21,22]. In some cases, however, clinical trials have failed, leading to the reevaluation or redesign of inhibitors. This review discusses some of interesting MAP kinase inhibitors used for cancer research. 

## 2. MAP Kinase Inhibitors in Cancer Research

SP600125 is a selective and reversible inhibitor of JNK kinases, which has an IC50 for JNK1, JNK2 = 40 nM, and JNK3 = 90 nM. It induces cell death selectively in undifferentiated thyroid cancer cell lines [23], reduces the viability of doxorubicin-resistant stomach cancer cells [24], sensitizes the multidrug-resistant KBV20C human oral squamous carcinoma cell line [25], enhances dihydroartemisinin-induced apoptosis in human lung adenocarcinoma cells [26], enhances transforming growth factor beta (TGF-β)-induced apoptosis in human cholangiocarcinoma cell line RBE [27], selectively kills p53-deficient human colon carcinoma cells in a mouse xenograft model [28], affects the regulation of the epithelial barrier function and cell shape during the remodeling of pancreatic cancer cells [29], and suppresses glioblastoma cells [30]. Apart from anticancer properties, this inhibitor is also used in inflammation research [31,32], and neuroprotection [33,34].

**Figure 2:**
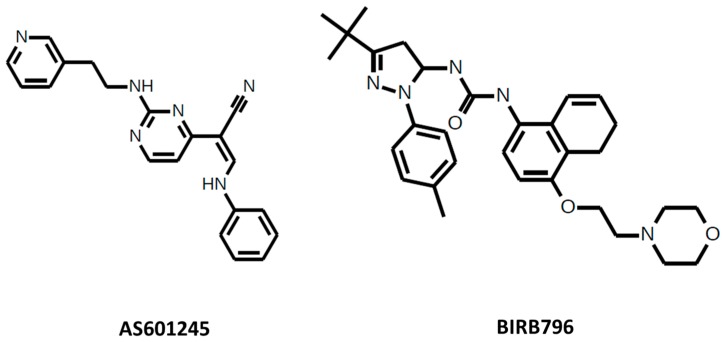
AS601245 and BIRB796.

*AS601245* is a cell-permeable JNK inhibitor (Figure 2). The IC50 for JNK1 = 150 nM, for JNK2 = 220 nM, and for JNK3 = 70 nM. AS601245 affects the proliferation of colon cancer cell lines [35] and decreases cell adhesion and migration via a decrease in the fibrinogen release in human colon cancer cells [36]. AS601245 also has an effect on leukemia by leading T-cell acute lymphoblastic leukemia cells to cell cycle arrest and apoptosis [37] and sensitizing promonocytic leukemia cells to arsenic trioxide-induced apoptosis [38]. In addition to cancer, AS601245 is also used in inflammation [39] and as antiviral agent [40,41].

*CC-401* is a specific inhibitor of JNK which has an IC50 for these kinase in a range of 25–50 nM. CC-401 in combination with oxaliplatin shows synergism in colon cancer cell lines SW620 and HT29 in vitro and in mouse xenografts [42]. It is also used for glomerulonephritis [43,44] and hepatic ischemia reperfusion injury [45,46].

Several other JNK inhibitors show promise in cancer cells: *AS602801* (IC50 for JNK1 = 80 nM, for JNK2 = 90 nM, and for JNK3 = 230 nM) inhibits cancer stem cells in vitro and in vivo [47], *D-JNKI-1* (IC50 for JNKs is 2.31 μM) reduces tumor growth in a mouse skin cancer model [48], and *BI-78D3* (IC50 for JNKs is 280 nM) sensitizes osteosarcoma to doxorubicin [49].

*SCIO-469* (Talmapimod) is a selective p38 inhibitor. The IC50 for p38α = 9 nM and for p38β = 90 nM. SCIO-469 enhances bortezomib-induced cytotoxicity against multiple myeloma cells [50], reduces multiple myeloma cell proliferation and adhesion [51], enhances the apoptosis of myeloma cells and inhibits tumor growth [52], and decreases tumor burden and angiogenesis in murine models of multiple myeloma [53,54]. In addition, it enhances the arsenic trioxide-dependent induction of apoptosis in chronic myelogenous leukemia or acute promyelocytic leukemia-derived cell lines [55]. This inhibitor is also used in phase II human clinical trials for the treatment of rheumatoid arthritis [56], myelodysplastic syndrome [57], and acute dental pain [58].

*BIRB-796* (Doramapimod) is a p38 inhibitor (Figure 2) which has an IC50 for p38α = 38 nM, for p38β = 65 nM, for p38γ = 200 nM, and for p38δ = 520 nM. BIRB-796 enhances cytotoxicity and inhibits paracrine tumor growth in multiple myeloma [59], enhances the efficacy of chemotherapeutic agents in multidrug resistance protein 1 (ABCB1) overexpressing oral epidermoid carcinoma cells [60], and enhances the antitumor effects of aurora kinase inhibitor VX680 in cervical cancer [61]. It is also used in inflammation research [62,63].

*LY2228820* (Ralimetinib) is a selective p38 inhibitor. The IC50 for p38α = 5.3 nM and for p38β = 3.2 nM. LY2228820 enhances bortezomib-induced cytotoxicity and inhibits osteoclastogenesis in multiple myeloma [64], produces significant tumor growth delay in multiple in vivo cancer models (melanoma, non-small cell lung cancer, ovarian, glioma, myeloma, breast) [65], and inhibits the Ras-related C3 botulinum toxin substrate 3 (Rac3)-induced cell invasion and migration of lung adenocarcinoma [66]. A Phase I clinical trial in patients with advanced cancer (colorectal, breast, sarcoma, non small cell lung, renal, pancreatic, melanoma, and ovarian) demonstrated acceptable safety, tolerability, and pharmacokinetics [67]. Although none of the patients had either full or partial remission, 19 (23.3%) patients had a stable disease within a median time of 3.7 months. That shows some promise using this inhibitor either as a single agent or in combination with chemotherapeutic agents; however, additional studies are required to find biomarkers that predict the clinical efficiency of LY2228820 for patients with advanced cancer. Another Phase I/II trial study of LY2228820 plus gemcitabine and carboplatin for platinum-sensitive ovarian cancer is still ongoing [68]. 

A couple other p38 inhibitors show promise in cancer cells: *VX-745* (IC50 for p38α = 10 nM and for p38β = 220 nM) inhibits multiple myeloma cell growth [69] and *PH-797804* (IC50 for p38α = 26 nM and for p38β = 102 nM) reduces tumor growth in colon tumor xenografts [70]. 

**Figure 3:**
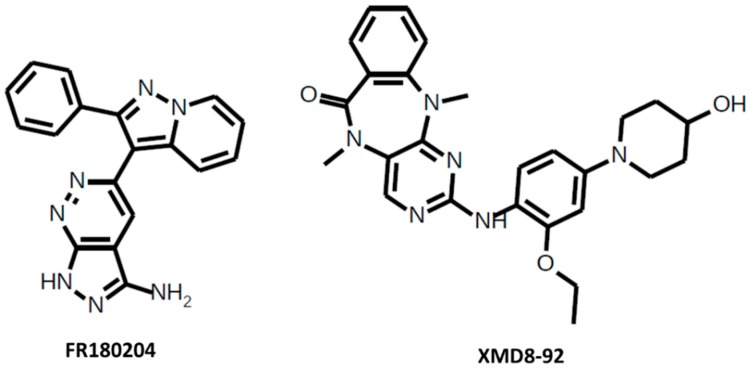
FR180204 and XMD8-92.

*FR180204* is a selective ERK1/2 inhibitor (Figure 3). The IC50 for ERK1 = 0.14 μM and for ERK2 = 0.31 μM. FR180204 attenuates mesothelioma cell proliferation [71], decreases cell viability colorectal cancer cell lines [72], decreases cell proliferation and increases apoptosis in colorectal cancer cells in combination with AKT inhibitor API-1 [73], and inhibits intestinal myofibroblast migration induced by KRAS-mutated colorectal cancer cells [74]. This inhibitor is also used in arthritis research [75]. 

*XMD8-92* is a selective ERK5 inhibitor (Figure 3), which has an IC50 for ERK5 = 300 nM. XMD8-92 blocks tumor cell proliferation in vitro and significantly inhibits tumor growth in a lung and cervical tumor xenograft model by 95% [76], causes cell cycle arrest in G2 phase in acute myeloid leukemia cells [77], inhibits pancreatic tumor xenograft growth [78], reduces proliferation, cell cycle progression, cell migration, and invasion in hepatocellular carcinoma cells, reduces tumor growth in xenografts [79], increases colon cancer cell sensitivity to 5-fluorouracil in a murine subcutaneous xenograft model [80], and impairs resistance to the combined inhibition of RAF proto-oncogene serine/threonine kinase B (BRAF) and MEK1/2 and the proliferation of resistant cells [81].

*SI113* is a selective SGK1 inhibitor, which has an IC50 for SGK1 = 600 nM. This inhibitor induces autophagy, apoptosis, and cell viability through endoplasmic reticulum stress in endometrial cancer cells [82], induces apoptosis and cytotoxic autophagy as well as increases the effects of radiotherapy and the response to oxidative stress in glioblastoma cells [83], blocks tumor progression in vitro and in liver hepatocellular carcinoma xenograft mice models, and synergizes with radiotherapy [84] (Table 1). 

## 3. Conclusions and Future Perspectives

In the past couple of decades, developments in the small-molecule MAP kinase inhibitor field have led to quite a number of marketed products with a diverse range of the inhibited targets. In addition, many more are still in development and/or improvement. The assortment of these inhibitors allows researchers to choose the most effective and appropriate methodologies suitable for the specific experiments. Preclinical studies both in cell lines as well as in proper animal models provide essential information for the design of clinical studies evaluating the improved efficiency of these agents. Clinical studies with MAP kinase inhibitors ought to determine which MAP kinase inhibitors are most effective for anticancer therapy. Many types of cancers can be targeted by inhibitors, as can be seen in this review. The particular use of MAP kinase inhibitors greatly depends on the genetic background and the precise signaling pathways that direct the cancerogenous properties of the cells in a given cancer type. 

Although there has been significant progress lately in the development of MAP kinase inhibitors, there remains much to be improved. Firstly, for the treatment of cancers, kinase inhibitors have to have high specificity in order to avoid off-target inhibition. However, keeping in mind that there are more than 510 kinases in our cells, it is not an easy task. Sequence similarities between kinases and especially between isoforms of the same family could make it difficult to design specific inhibitors. Peptide inhibitors or aptamers [85] could be a solution. *D-JNKI-1* is a good example showing that peptide inhibitors can go as far as clinical trials [86]. On the other hand, it is known that kinase inhibitors can inhibit other non-kinase targets. One good example is an NAD(P)H dehydrogenase 2 (NQO2), which can be inhibited by kinase inhibitors imatinib, nilotinib, TBB, and DMAT [87]. Therefore, more extensive research on off-target possibilities is needed. So far, after a new inhibitor is designed, it is common practice to test it on a panel of other kinases. However, kinase inhibitors are not tested against other proteins. Probably proteomics or similar high throughput techniques could help to achieve that. Then again, new approaches, such as computer-assisted, 3D structure-based approaches to generate new generations of kinase inhibitors, could be a solution for the future of MAP kinase inhibitors [87,88]. Structural insights into the distinctive inhibitor-kinase binding mechanisms have led to the discovery of several highly selective inhibitors [89,90,91]. Besides, in many cases, combination therapies using kinase inhibitors and chemotherapeutic agents or radiotherapy are even more encouraging than the use of these inhibitors as single agents. The achievements of combination therapies have already been shown both for MAP kinase (as mentioned in this review) and other kinases, such as CDKs, aurora kinase A (AURKA), tyrosine kinase, or multi-kinase inhibitors. Therefore, this field of both preclinical and clinical research should be further advanced. The other problem in kinase inhibitor therapies is the resistance of some tumors to these inhibitors. There are many pathways that lead to resistance and each requires a different solution. One of the problems could arise because of the overactivation of downstream effectors, the solution to which could be the inhibition of these factors. Activation of compensatory pathways could be another problem, possibly solved by dual inhibition. Mutations in target kinases could also render inhibitors insensitive, which again requires alternative inhibitors or at least combinatorial treatment. Some other problems, such as drug inactivation, multidrug resistance, or epigenetics, could be more problematic and less effectively solved. 

Interestingly, there are several alternative compounds which show some potential as MAP kinase inhibitors in cancer research. The natural compound Sulforaphane, extracted from cruciferous vegetables, suppresses ERK and AKT phosphorylation and induces apoptosis through G2/M phase arrest in osteosarcoma cells [92]. Metformin, a known diabetes type II drug, together with arsenic trioxide, which is known for acute myeloid leukemia treatment, suppresses intrahepatic cholangiocarcinoma cell proliferation via the regulation of AMP-activated protein kinase (AMPK), p38, ERK3, and mammalian target of rapamycin complex 1 (mTORC1) pathways [93]. RH1, a compound shown to inhibit the proliferation of several cancers, was also bioinformatically shown to potentially inhibit several kinases, including ERK2 [87]. 

In conclusion, the future seems to be bright for MAP kinase inhibitors and they definitely should not be dismissed.

## Figures and Tables

**Table 1:** The summary of small molecule inhibitors with their potential usages.

Ihibitor	Target	Potential Usages in Cancer
SP600125	JNK	stomach cancer [24], oral squamous carcinoma [25], lung adenocarcinoma [26], cholangiocarcinoma [27], colon carcinoma [28], pancreatic cancer [29], glioblastoma [30]
AS601245	JNK	colon cancer [35], leukemia [37,38]
CC-401	JNK	colon cancer [42]
SCIO-469	p38	multiple myeloma [50], leukemia [55]
BIRB-796	p38	multiple myeloma [59], oral epidermoid carcinoma [60], cervical cancer [61]
LY2228820	p38	melanoma, non-small cell lung cancer, ovarian cancer, glioma, myeloma, breast cancer [65], lung adenocarcinoma [66], phase I clinical trial in colorectal, breast, sarcoma, NSCLC, renal, pancreatic, melanoma and ovarian [67], phase I/II trial [68]
FR180204	ERK1/2	colorectal cancer [72]
XMD8-92	ERK5	lung cancer, cervical cancer [76], acute myeloid leukemia [77], pancreatic cancer [78], hepatocellular carcinoma [79], colon cancer [80]
SI113	SGK1	endometrial cancer [82], glioblastoma [83], hepatocellular carcinoma [84]

JNK: c-Jun N-terminal kinase; ERK: extracellular regulated kinase; SGK1: serum/glucocorticoid-regulated kinase 1; NSCLC: non small cell lung cancer.
